# Author Correction: High-power electrically pumped terahertz topological laser based on a surface metallic Dirac-vortex cavity

**DOI:** 10.1038/s41467-024-49563-9

**Published:** 2024-06-21

**Authors:** Junhong Liu, Yunfei Xu, Rusong Li, Yongqiang Sun, Kaiyao Xin, Jinchuan Zhang, Quanyong Lu, Ning Zhuo, Junqi Liu, Lijun Wang, Fengmin Cheng, Shuman Liu, Fengqi Liu, Shenqiang Zhai

**Affiliations:** 1 Laboratory of Solid-State Optoelectronics Information Technology, Institute of Semiconductors, Chinese Academy of Sciences, Beijing, China; 2 Center of Materials Science and Optoelectronics Engineering, University of Chinese Academy of Sciences, Beijing, China; 3 Division of Quantum Materials and Devices, Beijing Academy of Quantum Information Sciences, Beijing, China

**Keywords:** Quantum cascade lasers, Terahertz optics, Topological matter

Correction to: *Nature Communications* 10.1038/s41467-024-48788-y, published online 24 May 2024

The original version of this Article had an incorrect ORCID account 0009-0001-9834-9619 for Shenqiang Zhai, whose correct ORCID account is 0000-0003-3186-4461. The original article has been corrected.

