# Supplementary material for: High-power electrically pumped terahertz topological laser based on a surface metallic Dirac-vortex cavity
Source: Nat Commun. 2024 May 24;15:4431. doi: 10.1038/s41467-024-48788-y (PMC11126746; doi:10.1038/s41467-024-48788-y)
Supplement: Supplementary file 1 — Supplementary Information [file 41467_2024_48788_MOESM1_ESM.pdf]

Supplementary materials for "High-power electrically pumped terahertz topological laser based on a surface metallic Dirac-vortex cavity"

Junhong Liu[1,2,4], Yunfei Xu[1,2,4], Rusong Li[3], Yongqiang Sun[1,2], Kaiyao Xin[1,2], Jinchuan Zhang[1,2,*], Quanyong Lu[3,*], Ning Zhuo[1], Junqi Liu[1,2], Lijun Wang[1,2], Fengmin Cheng[1], Shuman Liu[1,2], Fengqi Liu[1,2,*] & Shenqiang Zhai[1,*]

[1]Laboratory of Solid-State Optoelectronics Information Technology, Institute of Semiconductors, Chinese Academy of Sciences, Beijing, China.

[2]Center of Materials Science and Optoelectronics Engineering, University of Chinese Academy of Sciences, Beijing, China.

[3]Division of Quantum Materials and Devices, Beijing Academy of Quantum Information Sciences, Beijing, China.

[4]These authors contributed equally: Junhong Liu, and Yunfei Xu.

* Corresponding author: zhangjinchuan@semi.ac.cn; luqy@baqis.ac.cn; fqliu@semi.ac.cn; zsqlzsmbj@semi.ac.cn;

S1. Cavity design theory

For the two-dimensional system, the effective continuum Hamiltonian takes the form:

$$\hat{H}_{eff}(k) = \begin{pmatrix} \hat{H}_{\uparrow}(k) & 0 \\ 0 & \hat{H}_{\downarrow}(k) \end{pmatrix} \tag{S1.1}$$

where $\hat{H}_{\uparrow}(k) = m(r)\hat{\sigma}_z + k_x\hat{\sigma}_x + k_y\hat{\sigma}_y$, $\hat{H}_{\downarrow}(k) = m(r)\hat{\sigma}_z - k_x\hat{\sigma}_x + k_y\hat{\sigma}_y$, k_i is the momentum and $\hat{\sigma}_i$ is Pauli matrices. Equation S1.1 supports an energy spectrum in the form:$E(k) = \pm\sqrt{m^2 + k^2}$.

A non-zero mass term $m(r)$ produces a gapped Dirac spectrum. The introduction of the mass term gaps the Dirac spectrum and thus allows confined solutions which gives rise to discrete localized states. The 2D topological cavity designed here is based on the zero-mode solutions of Dirac equations with mass vortices which was first proposed by Jackiw et al. The Kekulé modulation was introduced to construct mass vortices around the cavity for Jackiw-Rossi zero mode operation where the displacement vector m is defined by the following formula:

$$\mathbf{m} = m \;\; tanh\left(\frac{r}{R}\right) e^{iw(\theta_0 - \theta_r)} \tag{S1.2}$$

As defined, m represents the magnitude of displacement, r means the distance from primitive cell center O' to cavity center O, R represents the cavity vortex radius, w means the winding number which corresponds to the number of topological mid-gap modes, θ_0 is the initial relative phase of the of the three dotted lines in Fig. 2a (lines between three circles center and primitive cell centerO'), $\theta_r = atan\left(\frac{y}{x}\right)$ represents the angle between the center of the primitive cell and the center of the cavity as illustrated in Fig.2b. In order to ensure the single-mode operation of the laser, we have chosen the winding number of $w = +1$ in this article. Meanwhile, the maximum free spectrum range (FSR) can be achieved[1] when $R = 0$, which contributes to stable single-mode operation theoretically. Therefore, we chose $R = 0$ in this article where the formula (S1.1) can be simplified as follow:

$$\mathbf{m} = m \ \ e^{i(\theta_0 - \theta_r)} \tag{S1.3}$$

Considering the cavity parameter m, a larger m results in greater photonic bandgap and a larger FSR[2] which are conducive to higher surface emission power. But limited by the spacing between different sites, a too-large m can cause lattice deformation. Therefore, devices with different m are constructed.

S2. Effective refractive index calculation

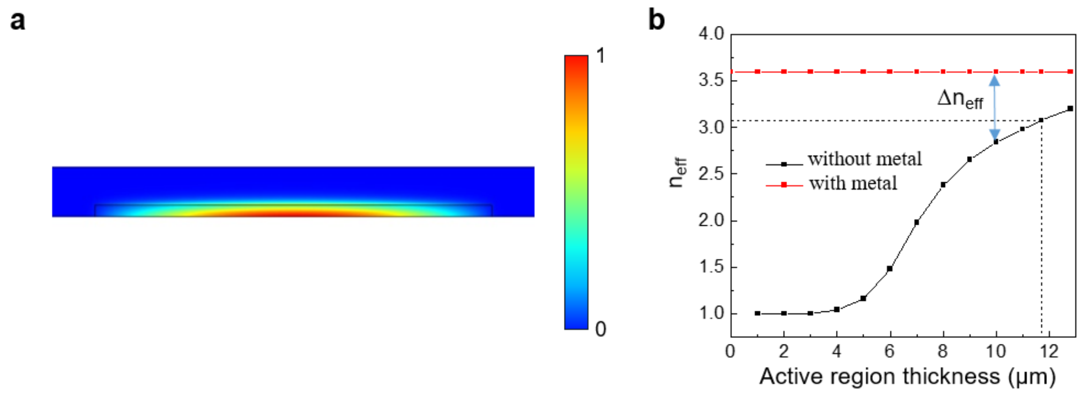

Fig. S1 | Effective refractive index calculation. a Simulation diagram and **b** Effective refractive index changes as the thickness of the active region.

Firstly, we calculate the effective refractive index of the device with non-etched active region for later calculations of the band diagram of the topological cavity based on the finite element method. As the thickness of the active region increases, the effective refractive index increases rapidly. The thickness of our active region is 11.7 μm. As shown in Fig. S1b, the effective refractive indexes of 3.08 and 3.6 are obtained for structures without and with metal covering, respectively. A large effective refractive index contrast can be achieved for a thinner active region layer. Although the decrease in the thickness of the active region will introduce reduced cascade periods and thus smaller gain, the large refractive index contrast can improve the surface emission efficiency, which may also result in high output power.

S3. SMDC TLs device performances under different temperatures

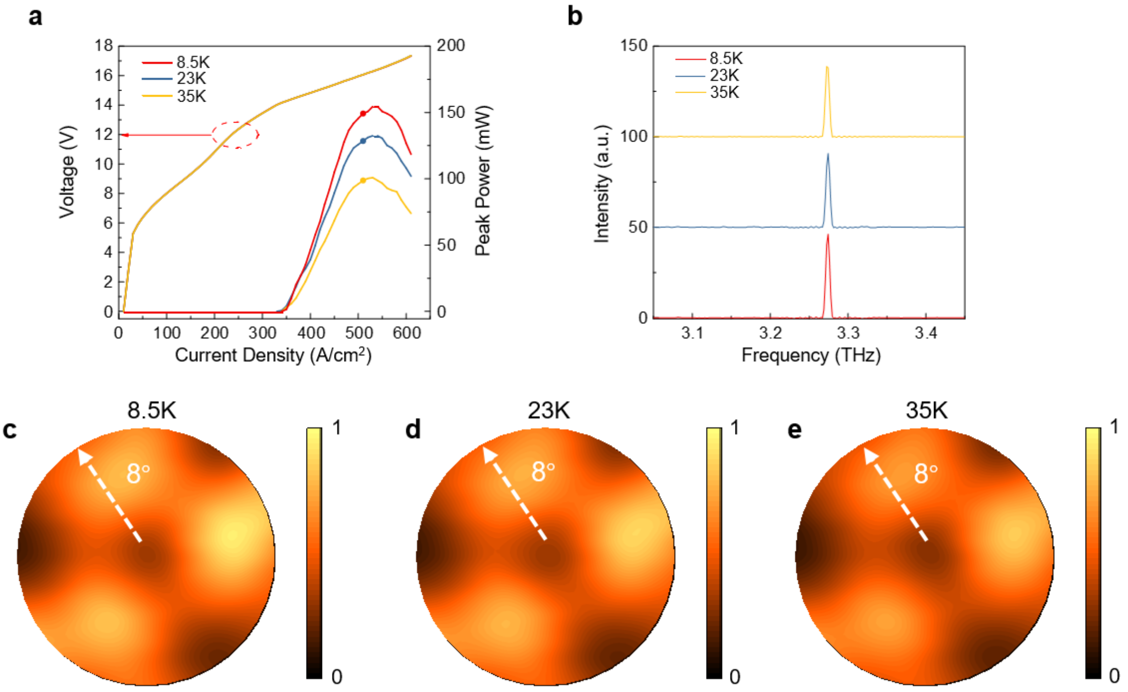

Fig. S2 | Performance of the SMDC device with $m = 0.18a$ and $a_0 = 30.5\mu m$ under different temperatures. a L-I-V results under different temperatures. **b** Lasing spectra under different temperatures. **c.d.e** Far-field patterns under different temperatures.

We also characterized the performances of the SMDC TLs under different temperatures, as shown in Fig. S2. With the increasing temperatures from 8.5 K to 35 K, the devices still maintain robust single-mode operations and C_{3V} symmetric far-electric fields, which indicate the operating robustness of the devices with different temperatures.

S4. The effects of the absorbing boundary condition on lasing properties

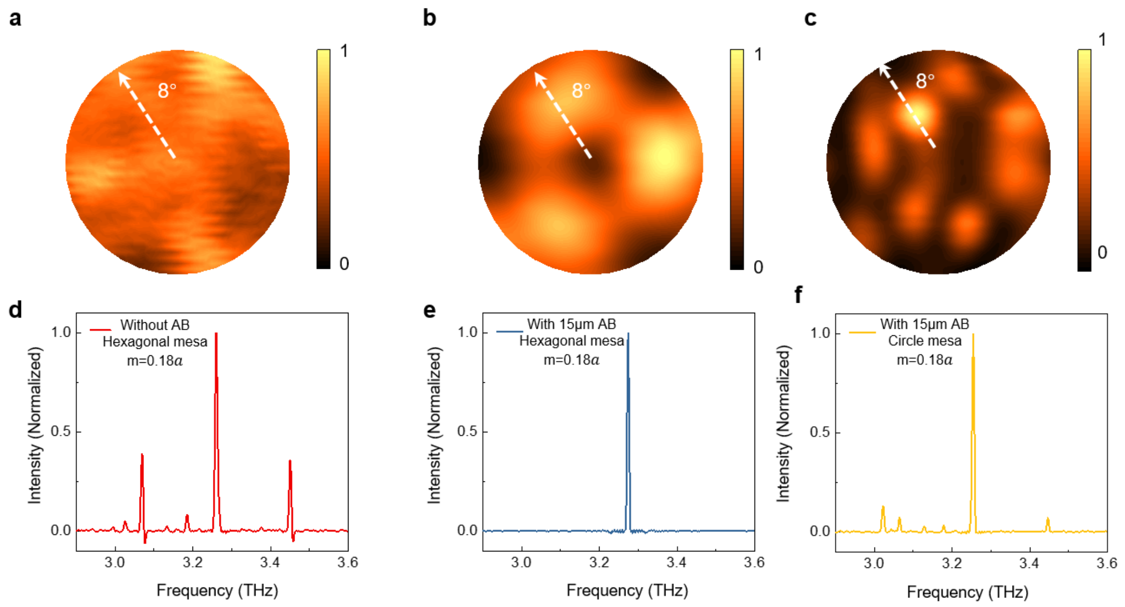

Fig. S3 | The effects of the absorbing boundary condition on lasing properties. a,d Far-field pattern and lasing spectrum of the hexagonal mesa cavity device without absorbing boundary**. b,e** Far-field and spectrum of hexagonal mesa cavity device with 15 μm wide highly doped absorbing boundary. **c,f** Far-field and spectrum of circle mesa device C with 15 μm wide highly doped absorbing boundary.

To study the effects of the absorbing boundary condition on lasing properties, we fabricated devices with different boundary condition for comparison. Lasing spectra and far-field patterns of the devices with different boundary conditions and parameter of $m = 0.18a$ and $a_0 = 30.5\mu\text{m}$ are shown in Fig. S3. As is show in Fig. S3, the absorption boundary design has played an important role in the realization of highly robust single-mode lasing. The device without absorption boundary can't operate stably in topological mode while the device with absorption boundary demonstrates the robust operation of topological mode, which is illustrated by both the lasing spectra and the far-field patterns. This can be attributed to the increasing loss of the whispering gallery modes due to the introduction of the absorption boundary. Besides, in Fig. S3c and S3f, the circle mesa cavity with circular absorption boundary device demonstrates multi-mode operation and the far-field also does not satisfy the C_{3v} symmetry, since the whispering-gallery mode will have a lower loss for a circular boundary.

S5. Comparison of different types of devices

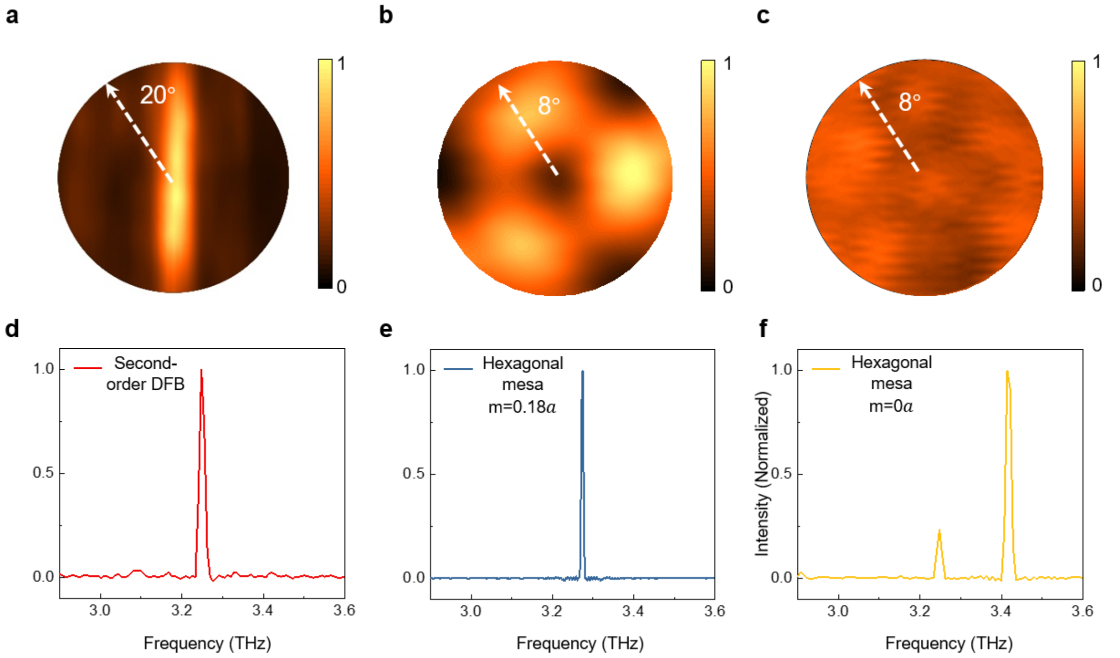

Fig. S4 | Far-field patterns and lasing spectra for different types of devices. a,d Far-field pattern and lasing spectrum of the second-order DFB device. **b,e** Far-field pattern and lasing spectrum of the topological device of hexagonal mesa with $m = 0.18a$ and $a_0 = 30.5$μm. **c,f** Far-field pattern and lasing spectrum of the trivial photonic crystal device with $m = 0a$ and $a_0 = 30.5$μm.

As is shown in Fig. S4a, although the second-order DFB device demonstrates single-mode operation, the far-field demonstrates a long strip pattern. To maintain the fundamental mode operation, the ridge width of the second-order DFB device is limited which results in a large far-field divergence angle. The device with $m = 0a$ demonstrates multi-mode operation. This can be attributed to the unoptimized design of this photonic crystal structure, which results in a band-edge structure with many multiple modes having the similar gain condition for lasing. However, from another perspective, this demonstrates the important role of the introduction of the vortex cavity for the single-mode operation. The photonic crystals device can also realize a large-threshold gain difference with carefully design[3,4]. Compare with photonic crystal device lasing on the band edge modes, the topological cavity device introduces an approach to obtain stable mid-gap single-mode operation, which gives a large FSR

and threshold margin simultaneously.

S6. Calculation of loss and vertical radiative efficiency of the cavity modes

The loss of the cavity modes and vertical radiative efficiency are calculated using the three-dimensional (3D) full-wave finite element method. For the calculations, we have taken the device with $m = 0.18a$ and $a_0 = 30.5\mu m$ as the example.

The calculated Q factors for different modes are shown in Figure S5. The photon loss rate γ_r due to the vertical radiation of the laser is estimated by extracting the time-averaged integrated power flow through the open air domains and normalizing it with respect to the resonator energy in the 3D simulation[5]:

$$\gamma_r = \frac{\Phi}{E_{res}} = \frac{\int_A (E \times H) \cdot \hat{n} dS}{\int_V (\epsilon |E|^2 + |H|^2/\mu) dV} \quad (R.1)$$

Where E and H represent the electric and magnetic fields respectively, $\hat{n}$ represents the unit normal vector of the circle air domains, ϵ the dielectric constant and μ the permeability. The corresponding quality factors have been derived from the relation $Q_{vertical} = v/\gamma_r$, where v is the eigenfrequency of the topological mode. And the radiative out-coupling efficiency η_r is assumed to be proportional to $\frac{Q_{total}}{Q_{vertical}}$ where:

$$Q_{total} = \left(\frac{1}{Q_{inplane}} + \frac{1}{Q_{vertical}} \right)^{-1} \quad (R.2)$$

Based on the 3D simulation, a photon loss rate of $\gamma_r \approx 28.5$ GHz from integral results through equation (R.1) is obtained, which corresponds to $Q_{vertical} = 140$. Considering the calculated $Q_{total} = 96$ from the 3D simulation, an in-plane quality factor $Q_{inplane} = 320$ is obtained. As a result, an out-coupling efficiency of $\eta_r \approx 68.5\%$ is estimated. This result is obtained without considering the waveguide loss.

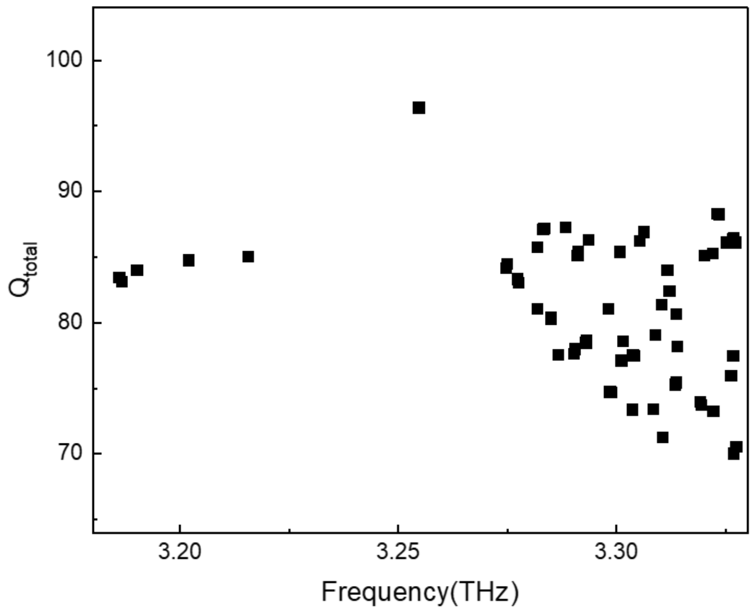

Figure S5. 3D simulation of the Q factors for different modes

Next, we use the experimental results of the F-P device from the same wafer as the topological cavity device to modify the theoretical calculation considering the waveguide loss. For the F-P device with 2 mm cavity length and 100 μm ridge width, we have $\alpha_m = \frac{1}{2L}\ln\left(\frac{1}{R_1 R_2}\right) = \frac{1}{L}ln\left(\frac{1}{R}\right)$, and the facet reflectivity for metal-metal waveguide structure with waveguide width of 100 μm and active region thickness of 11.7 μm around 3.2 THz is about 80%[6]. Therefore we obtain $\alpha_m \approx 1.1cm^{-1}$. Meanwhile, the total optical loss coefficient α_{total} for F-P plasmonic QCLs operating in the wavelength range of 3~4 THz[6-8] has been experimentally measured in the range of $10{\sim}15cm^{-1}$. Considering $\alpha_{total} = \alpha_m + \alpha_w$, where α_w is waveguide loss, we take the $\alpha_{total} = 12.5cm^{-1}$ and $\alpha_w = 11.4cm^{-1}$ is obtained.

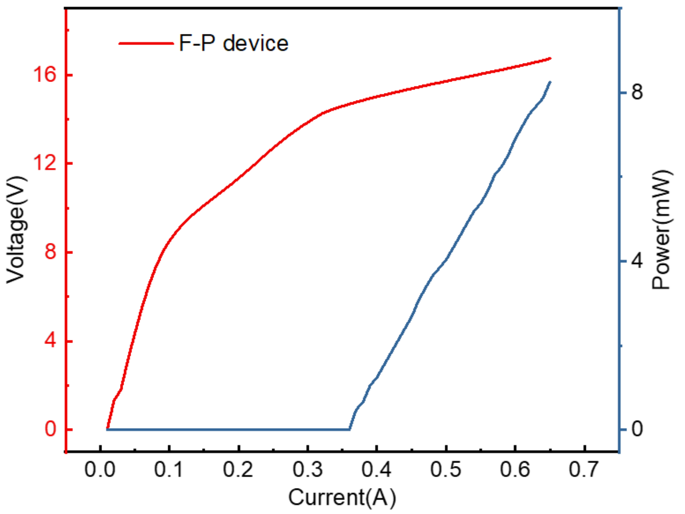

Figure S6. L-I-V curves of the F-P device with 2 mm cavity length and 100 μm ridge width.

Figure S6 shows the L-I-V curves of the F-P device with slope efficiency of $28\ mW/A$. In theory, the slope efficiency of the F-P device can be written as

$$\eta_{slope\_FP} = \eta_i \frac{N\hbar\omega}{e} \eta_{rad\_FP} = \eta_i \frac{N\hbar\omega}{e} \frac{\alpha_m}{\alpha_m + \alpha_w} \qquad (R.3)$$

Meanwhile, as for the topological cavity device, the total optical loss coefficient and the slope efficiency can be written as

$$\alpha_{total} = \alpha_{\perp} + \alpha_{\parallel} + \alpha_w \qquad (R.4)$$

$$\eta_{slope\_Topo} = \eta_i \frac{N\hbar\omega}{e} \eta_{rad_{Topo}} = \eta_i \frac{N\hbar\omega}{e} \frac{\alpha_{\perp}}{\alpha_{\perp} + \alpha_{\parallel} + \alpha_w} \qquad (R.5)$$

Where $\alpha_{\perp}$ represent optical loss coefficient due to the vertical radiation, $\alpha_{\parallel}$ is the in-plane mirror loss which can be obtained from $\alpha = \frac{2\pi * n_{eff}}{\lambda * Q}$. Hence $\alpha_{\perp}$ ($Q_{vertical}$=140) and $\alpha_{\perp} + \alpha_{\parallel}$ (Q_{total} =96.7) are calculated to be $17.5 cm^{-1}$ and $25.5 cm^{-1}$ respectively. Therefore, after taking the waveguide loss α_w in to consideration, we obtain $\eta_{rad\_Topo} = \frac{17.5}{(25.5+11.4)} = 0.474$ for the SMDC TL device with $m = 0.18a$ and $a_0 = 30.5$ μm. Meanwhile, the slope efficiency η_{slope_Topo} $151 mW/A$ is obtained from the P-I-V curve in Fig. 3b experimentally. Therefore, we have the theoretically calculated slope efficiency ratio for the topological and FP devices $\frac{\eta_{rad\_Topo}}{\eta_{rad\_FP}} = \frac{0.474}{1.1/12.5} = 5.386$, which is in excellent agreement with the experimental data $\frac{\eta_{slope\_Topo}}{\eta_{slope\_FP}} = \frac{151}{28} = 5.393$. This verifies the reliability of the numerical simulation. This

relatively high outcoupling efficiency originates from the coherent constructive emission over the entire lattice area. Table S1 compared our results with other typical high-power surface-emitting THz QCL reported previously. Clearly, our device exhibits fairly competitive radiation loss and efficiency compared with devices based on different photonic designs, providing the huge potential for high output power. For the absolute output power of these devices, the epitaxial material used in the device fabrications is another critical factor, which may introduce the inconsistency between the radiation efficiency and output power. Therefore, enough gain provided by the surface metal cavity design and large vertical radiation loss and efficiency introduced by the topological photonics design result in the increase of the output power of our device together.

Table S1. Radiation loss and efficiency for different single-chip THz surface emitting QCLs

Physics mechanism	Radiation loss	Radiation efficiency η_{rad}
Graded gratings[9]	$11cm^{-1}$	≈34%
Hybrid second-and fourth-order gratings[10]	$6cm^{-1}$	≈30%(1)
Photonic quasi-crystal[5]	$2.7cm^{-1}$(2)	≈16%
Topological mid-gap mode (This Work)	$17.5cm^{-1}$	≈47.4%

(1) Estimated with the typical parameters from ref [6-8]

(2) Calculated from the Q factor

S7. Power performance of devices with different sizes

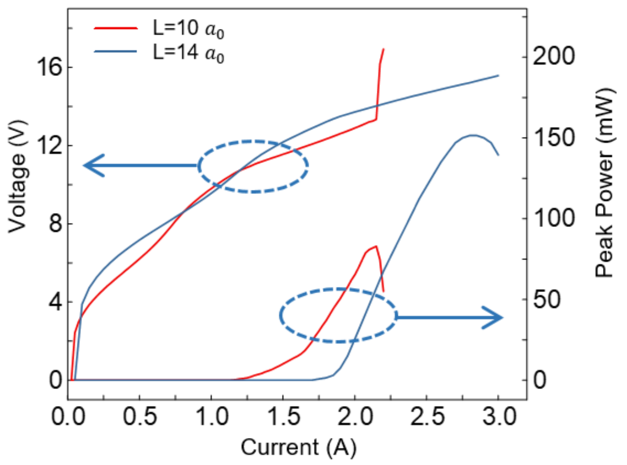

Fig. S7 | Power performance of devices with different sizes.

As shown in Fig. S7, the output power of the device with L of $14a_0$ is nearly twice the power of the device with L of $10a_0$, which demonstrates nearly linear power upscaling with increasing sizes. In further, the device can be made much larger for higher output power. Due to the large free spectral range (FSR) of the topological mid-gap mode, stable single mode operation is expected with larger device size. In further, smaller divergence angle will be obtained with increasing device size. However, the biggest challenge for large-area device is the heat dissipation issue of the device, nevertheless, this issue could be potentially addressed by thinning the thickness of the active region accordingly.

S8. Far-field testing optical path

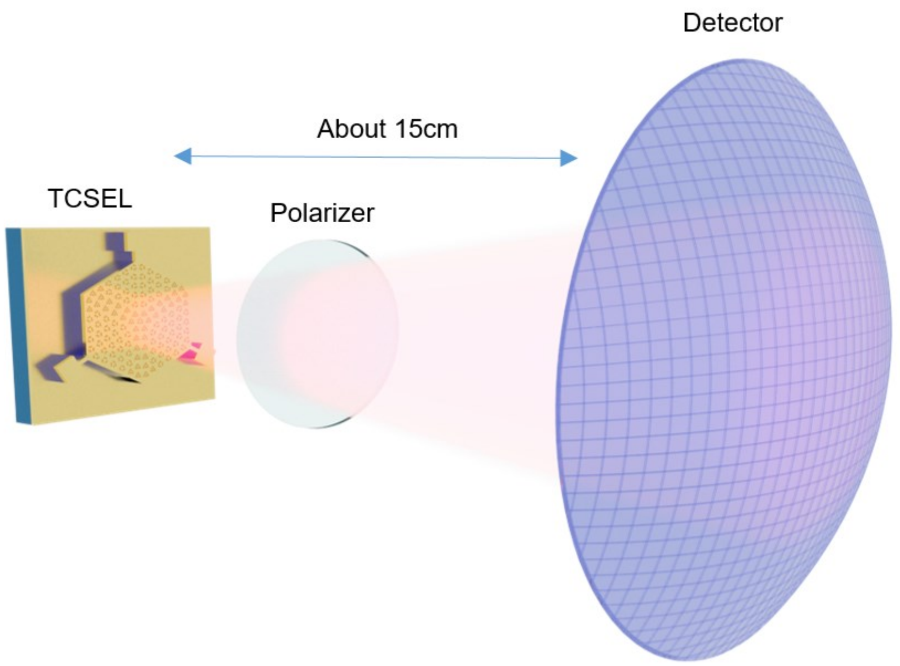

Fig. S8 | Schematic diagram of the far-field testing optical path

As illustrated in Fig. S8, the far-field properties of the devices were directly measured by a Golay cell detector scanning on the curve of a sphere with a radius around 15 cm from the device. For the polarized properties measurement, one linear polarizer was added to filter the cross-polarized components during the far-field intensity scanning. Through changing the direction of the linear polarizer, the polarized far-field properties were measured.

S9. Polarized far fields and lasing spectra of devices with phase modulation

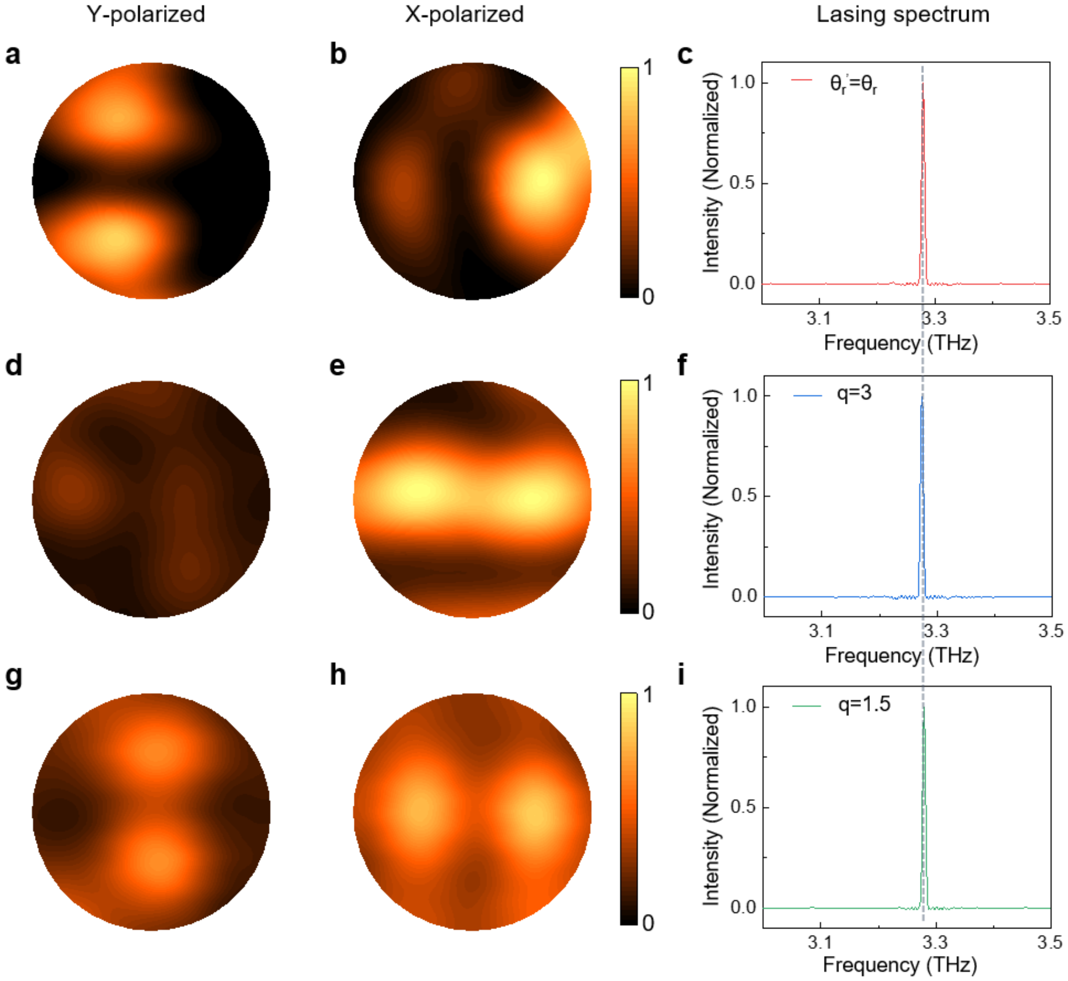

Fig. S9 | Polarized far-field patterns and lasing spectra of devices with various phase modulation conditions. a,b,c Polarized far-field pattern and lasing spectrum without phase modulation. **d,e,f** Polarized far-field pattern and lasing spectrum with phase modulation parameter $q = 3$. **g,h,i** Polarized far-field pattern and lasing spectrum with phase modulation parameter $q = 1.5$.

As is shown in Fig. S9, the devices with phase modulation can maintain the properties of vortex polarization while the symmetry of the far-field patterns changes from C_3 to double-lobed beam ($q = 3$) and doughnut beam ($q = 1.5$). In further, the lasing spectra also remain the same as that of device without phase modulation. These results shows that the symmetry of the output far-field can be tuned through phase modulation with topological lasing properties unchanged.

Supplementary References

1. Gao, X. et al. Dirac-vortex topological cavities. Nat. Nanotechnol. 15, 1012-1018 (2020).
2. Yang, L. et al. Topological-cavity surface-emitting laser. Nat. Photon. 16, 279-283 (2022).
3. Masahiro Yoshida et al. High-brightness scalable continuous-wave single-mode photonic-crystal laser. *Nature* 618, 727–732 (2023)
4. Contractor, R. et al. Scalable single-mode surface-emitting laser via open-Dirac singularities. *Nature* 608, 692–698 (2022).
5. Vitiello, M.S. et al. Photonic quasi-crystal terahertz lasers. *Nat Commun* **5**, 5884 (2014).
6. Kohen, S., Williams, B.S. & Hu, Q. Electromagnetic modeling of terahertz quantum cascade laser waveguides and resonators. *Journal of Applied Physics* **97** (2005).
7. Burghoff, D., Chan, C.W.I., Hu, Q. & Reno, J.L. Gain measurements of scattering-assisted terahertz quantum cascade lasers. *Applied Physics Letters* **100** (2012).
8. Jin, Y., Reno, J.L. & Kumar, S. Phase-locked terahertz plasmonic laser array with 2 W output power in a single spectral mode. *Optica* **7**, 708-715 (2020).
9. Xu, G. et al. Efficient power extraction in surface-emitting semiconductor lasers using graded photonic heterostructures. *Nat. Commun.* **3**, 952 (2012).
10. Jin, Y. et al. High power surface emitting terahertz laser with hybrid second- and fourth-order Bragg gratings. *Nat. Commun.* **9**, 1407 (2018).
